# Effectiveness of prior intra-articular corticosteroid injection in elderly patients with knee osteoarthritis undergoing progressive resistance training: a randomized controlled trial

**DOI:** 10.1186/s42358-025-00452-9

**Published:** 2025-04-28

**Authors:** Christine Brumini, Rita Nely Vilar Furtado, Anamaria Jones, Raphael Vilela Timóteo da Silva, Jamil Natour

**Affiliations:** Universidade Federal de Sao Paulo / Escola Paulista de Medicina (UNIFESP/EPM) – Disciplina de Reumatologia, Rua dos Otonis, 863 - Vila Clementino, Sao Paulo, SP CEP 04025-002 Brazil

**Keywords:** Osteoarthritis, Knee, Aged, Resistance training, Intra-articular injections

## Abstract

**Objective:**

To evaluate the effectiveness of intra-articular injections (IAIs) with triamcinolone hexacetonide (TH) combined with a progressive resistance exercise program (PREP) in improving pain, function, muscle strength, and quality of life in elderly patients with knee osteoarthritis (OA).

**Methods:**

Fifty-nine elderly individuals with knee OA were randomized into three groups: IAI with TH (IAI-TH) + PREP, IAI with saline solution (IAI-SS) + PREP, and IAI with placebo + PREP. The IAIs were administered once, one week before starting PREP, which was performed twice weekly for 12 weeks. Outcomes assessed at baseline and at 2, 6, and 12 weeks post-IAI included pain (Numerical Pain Scale - NPS), swelling, function (Western Ontario and McMaster Universities Osteoarthritis Index - WOMAC), quality of life (Short Form-36 - SF-36), performance tests (Six-Minute Walk Test − 6MWT, Timed Up and Go Test - TUGT, Short Physical Performance Battery - SPPB), and muscle strength (one-repetition maximum test − 1RM). Due to the COVID-19 pandemic, only 15 participants per group completed the study protocol.

**Results:**

All groups showed significant intragroup improvements over time in pain, function, muscle strength, and quality of life. However, no statistically significant differences were found between the groups for any of the assessed outcomes. The bodily pain domain of the SF-36 and analgesic consumption were the only measures showing differences over time.

**Conclusion:**

The combination of IAI-TH and a 12-week PREP (twice weekly) was not superior to IAI-SS or placebo combined with the same PREP in improving pain, function, or quality of life in elderly patients with knee OA. These findings highlight the role of exercise as a key therapeutic strategy, regardless of prior IAI. Future studies with larger sample sizes and long-term follow-ups are needed to better assess the role of intra-articular corticosteroid injections in OA rehabilitation.

**Clinical trial number:**

ensaiosclinicos.gov.br (RBR-556md5g). Registered 27 October 2022.

## Background

Osteoarthritis (OA) is the most prevalent osteoarticular disease and a leading cause of physical disability [1]. Its incidence increases with population aging and rising obesity rates, with the knee being the most commonly affected joint [2].

Current guidelines for knee OA divide treatment into pharmacological, non-pharmacological, and surgical approaches. Despite its high prevalence, OA remains a condition with limited pharmacological treatment options, making non-drug interventions the primary recommendation for disease management [3–5]. Exercise is the key recommendation for non-pharmacological treatment in OA patients (5–6), with systematic reviews indicating significant reductions in pain, improved function, and enhanced quality of life [7]. Studies suggest that exercise programs, particularly supervised and progressive resistance training, are the most effective interventions for managing OA symptoms [8, 9].

Intra-articular injections (IAIs) with corticosteroids (CEs) are commonly used in OA treatment for short-term pain relief (4–5, 10). Among corticosteroids, triamcinolone hexacetonide (TH) is one of the most frequently employed (11–12). The effectiveness of combining resistance exercise with IAI-TH in knee OA patients has been explored in prior studies. A pilot study by Parfitt et al. [13] compared IAI-TH alone versus IAI-TH followed by exercise, but due to the small sample size, no significant differences were found. Henriksen et al. [14] evaluated a 12-week supervised exercise program in patients receiving IAI-TH or saline, finding no significant differences between groups in terms of pain and function improvement. Another study by Guvendi et al. [15] analyzed a six-month home-based exercise program comparing multiple IAI therapies, showing inferior outcomes for corticosteroids compared to platelet-rich plasma (PRP).

Given that IAI-SS has demonstrated therapeutic effects (16–17), the lack of an appropriate control may have influenced the findings of previous studies. This study aimed to evaluate whether combining a progressive resistance exercise program with a prior corticosteroid IAI would enhance its effectiveness. We hypothesized that reducing knee inflammation before initiating exercise could potentiate its benefits.

## Methods

### Study design

This was a three-arm, double-blind, randomized, placebo-controlled trial conducted over 12 weeks, from December 2018 to March 2020.

### Participants, therapists, and study centers

Fifty-nine patients were recruited from the rheumatology outpatient clinic of the Rheumatology Department at the Universidade Federal de São Paulo, Brazil.

### Inclusion criteria

Participants were eligible if they met the following criteria:

- Both genders, aged ≥ 60 years.
- Diagnosis of knee osteoarthritis (OA) based on the American College of Rheumatology (ACR) criteria [18].
- Pain intensity between 4 and 8 cm on the Numerical Pain Scale (NPS) [19].
- Radiographic OA classification of grade II or III according to the Kellgren & Lawrence (KL) system [20].
- Symptoms persisting for more than three months.
- Stable medication regimen for at least three months before enrollment.

### Exclusion criteria

Patients were excluded if they presented:

- Diagnosis of inflammatory arthritis, gout, pseudogout, fibromyalgia, psychiatric disorders, or decompensated cardiovascular or neurological conditions affecting the lower limbs.
- Uncontrolled diabetes mellitus (DM) or systemic arterial hypertension (SAH).
- Physical therapy or acupuncture in the past three months.
- Initiation or modification of a regular physical activity routine or use of assistive walking devices within the last three months.
- Skin lesions preventing intra-articular injections (IAI).
- Prior IAI in the affected knee within three months or in any other joint within one month.
- Severe clotting disorders or lower extremity surgery in the previous six months.
- Suspected bacterial infection of any kind.
- Cognitive impairments preventing comprehension or adherence to the study protocol.

The study was approved by the institutional ethics committee, and all participants provided written informed consent (CAAE: 56545416.6.0000.5505). The trial was registered in the Brazilian Clinical Trials Registry (ensaiosclinicos.gov.br, RBR-556md5g), and this manuscript follows the CONSORT guidelines [21].

### Randomization, treatment allocation, and blinding

An electronically generated randomization list, created by a blinded statistician, was used to allocate patients into one of three groups:

- G1 (IAI/CE + PREP): Intra-articular injection of corticosteroid (CE) followed by a progressive resistance exercise program (PREP).
- G2 (IAI/SS + PREP): Intra-articular injection of saline solution (SS) followed by the same PREP.
- G3 (IAI/placebo + PREP): Placebo intra-articular injection (needle insertion without medication administration) followed by the same PREP.

Opaque, sealed envelopes were used to ensure allocation concealment. A researcher not involved in the study performed the randomization.

The only unblinded personnel was the rheumatologist administering the IAI. The physiotherapist conducting the PREP, the study participants, and the outcome assessor remained blinded to group allocation. To ensure blinding, syringes were prepared by the unblinded rheumatologist outside the participants’ view.

Baseline assessments were conducted before randomization and IAI administration. The PREP started one week after the IAI and continued for 12 weeks. All participants followed the same exercise protocol.

### Intervention

#### Intra-articular injection

- G1 (IAI/CE + PREP) received 3 mL (60 mg) of triamcinolone hexacetonide (TH, 20 mg/mL), followed by PREP.
- G2 (IAI/SS + PREP) received 3 mL of 0.9% saline solution, followed by PREP.
- G3 (IAI/placebo + PREP) received a placebo injection (needle insertion and withdrawal without fluid administration), followed by PREP.

The selection of triamcinolone hexacetonide concentration and volume was based on previous studies demonstrating its efficacy in intra-articular corticosteroid therapy for knee OA [22, 23]. The 60 mg dose in a 3 mL volume ensures appropriate joint dispersion and an optimal safety profile. Additionally, the 3 mL volume for saline and placebo injections was standardized to ensure consistency across groups and minimize bias.

Studies suggest that saline solution may have a therapeutic effect, supporting its inclusion as a control in this study. Although we have addressed the rationale for using saline as a control, we acknowledge that its potential therapeutic effects may raise concerns. To clarify this choice, we have now further detailed its role in the Methods section, citing meta-analyses that support its use as an appropriate comparator in intra-articular injection studie [24, 25]. The placebo injection was designed to replicate the intervention procedure while eliminating potential biological effects.

All IAIs were administered by a rheumatologist with 25 years of experience in interventional rheumatology. The accuracy of this rheumatologist’s blind intra-articular knee injections was previously validated at 100% in a study conducted by our group [26].

The most symptomatic knee was selected for the IAI, with patients positioned in a supine posture and the lower limb extended. The injection site was 2 cm from the superolateral angle of the patella, with the knee in slight eversion, using a 40 × 8 mm needle. In groups G1 and G2, a separate syringe containing 2% lidocaine (without vasoconstrictor) was used for local anesthesia when needed [22]. For knees with joint effusion, arthrocentesis was performed before injection. In the placebo group (G3), neither arthrocentesis nor medication injection was performed. All procedures were conducted under sterile conditions. Patients were shielded from visualizing the procedure to maintain blinding.

Each participant received only one IAI at baseline. No additional OA treatments were permitted during the study. Post-injection, patients were advised to rest the joint for 48 h, keep a bandage on the knee, and use analgesics as needed.

### Progressive resistance exercise program

**Fig. 1:**
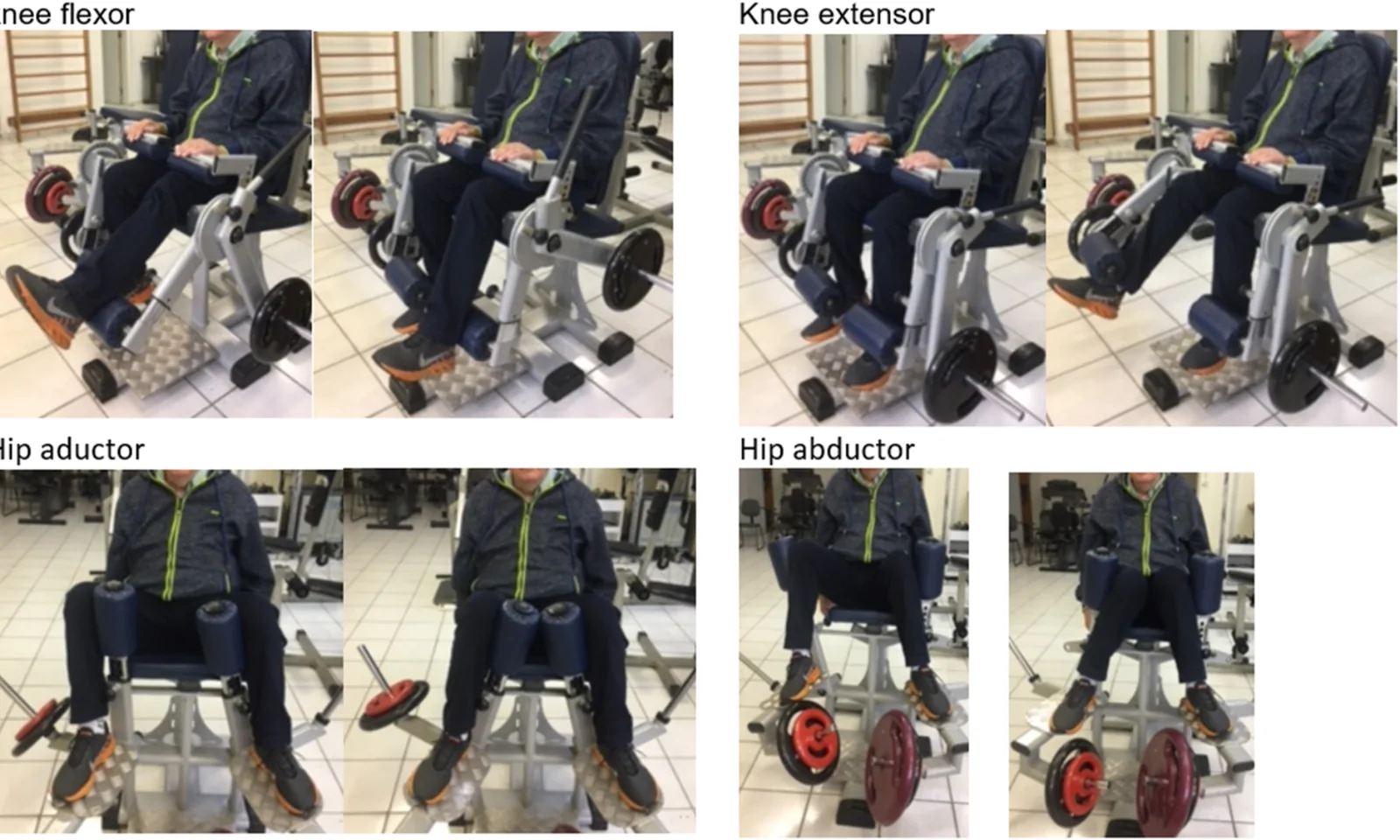
Exercises performed in the PREP

The exercise program was previously described in a prior trial [23]. In summary, it began with a 10-minute warm-up on a stationary bicycle, followed by four lower-limb muscle-strengthening exercises (Fig. 1). The program started one week after IAI and was conducted twice per week for 12 weeks, totaling 24 training sessions per participant. Patients who missed a session were encouraged to reschedule it within the same week. Attendance at each session was systematically recorded.

### Rescue medication

All participants had access to paracetamol 500 mg as rescue medication, to be taken as needed. Each patient was provided with a daily log to record their medication use over the 12-week follow-up period. The physiotherapist monitored and recorded the number of tablets consumed at each evaluation time point (T2, T6, and T12). No additional analgesic medications were prescribed as part of the study protocol.

### Outcomes

Evaluations were conducted by a blinded assessor at baseline (T0, before the IAI), and at 2 (T2), 6 (T6), and 12 (T12) weeks post-baseline. The following demographic and clinical data were collected at baseline: radiological KL classification, gender, ethnicity, duration of OA, affected side, comorbidities, weight, height, and living situation.

#### Primary outcome

- Pain was assessed using the Numerical Pain Scale (NPS), ranging from 0 cm (no pain) to 10 cm (worst imaginable pain) [19], evaluating pain at rest and during movement in the affected knee.

#### Secondary outcomes

- Joint swelling was measured by assessing knee circumference (cm) using a flexible measuring tape (150 cm), with measurements taken immediately proximal to the patella [24].
- Function was evaluated using different tools:
  - The WOMAC questionnaire, assessing pain, stiffness, function, and total score, with values ranging from 0 to 96, where higher scores indicate worse function [25, 26].
  - The 6-Minute Walk Test (6MWT) measured the distance covered over six minutes on a flat surface, with greater distances indicating better function [27].
  - The Timed Up and Go Test (TUGT) assessed the time taken (in seconds) to rise from a standard chair, walk 3 m, turn, return, and sit down, where lower times indicated better function [28].
  - The Short Physical Performance Battery (SPPB) was used to evaluate lower-limb physical performance in older adults, consisting of static balance (holding three different positions for 10 s each), gait speed over 4 m, and a chair stand test (standing and sitting five times consecutively). The total score ranged from 0 to 12, with higher scores indicating better physical performance [29].
- Muscle strength was assessed using the one-repetition maximum (1-RM) test, which determined the maximum load an individual could lift in a single attempt. Each participant had up to five attempts to determine their 1-RM for knee extensors, knee flexors, hip adductors, and hip abductors (Fig. 1) [30].
- Quality of life was measured using the SF-36 questionnaire, which evaluated eight domains (physical functioning, physical role limitations, bodily pain, general health, vitality, social functioning, emotional role limitations, and mental health). Scores ranged from 0 to 100, where higher scores indicated better quality of life [31].
- Patient satisfaction was assessed using a Likert scale with five response options: (1) I feel much better, (2) I feel a little better, (3) I feel the same as before, (4) I feel a little worse, and (5) I feel much worse [32].
- Medication consumption was tracked by evaluating the number of analgesic (acetaminophen 500 mg) or NSAID pills used at each evaluation time point. At T0, participants received a printed log covering the 12-week follow-up period, where they recorded their daily intake of analgesics. A blinded evaluator collected medication data at each assessment time point, starting at T2.

### Data analysis

Sample size calculation was based on a repeated-measures analysis of variance (ANOVA), measured five times across three groups, using NPS scores with a standard deviation (SD) of 2 cm as the main parameter. To detect a minimum effect size of 2 cm in the NPS (0–10 cm scale) with a 5% (α) error and 20% (β) error, the estimated minimum required sample was 47 patients per group. To account for a potential 10% dropout rate, the final target sample size was set at 52 patients per group.

Descriptive statistics were used to analyze the demographic characteristics of the sample, with means and SDs reported for continuous variables and frequencies and percentages for categorical variables. The Chi-square test, Kruskal-Wallis test, and repeated-measures ANOVA were applied to assess the baseline homogeneity of the groups. Additionally, ANOVA and MANOVA (multivariate analysis of variance) were used for repeated measures to evaluate changes in group behavior over time.

To enhance statistical reporting, 95% confidence intervals (CIs) were prioritized over p-values for intragroup comparisons. This decision aligns with current best practices in statistical analysis, as CIs provide a more comprehensive representation of the effect size and direction of change, reducing the reliance on arbitrary significance thresholds. The primary statistical focus was the interaction P-value, which indicates differences between the groups over time. This approach is in accordance with recent recommendations emphasizing the importance of effect estimation and interval estimation in clinical research, rather than binary statistical significance testing alone.

An intent-to-treat (ITT) analysis was performed for cases in which participants missed an evaluation, with their most recent available data carried forward. The level of statistical significance was set at *p* < 0.05 for all between-group comparisons.

## Results

**Fig. 2:**
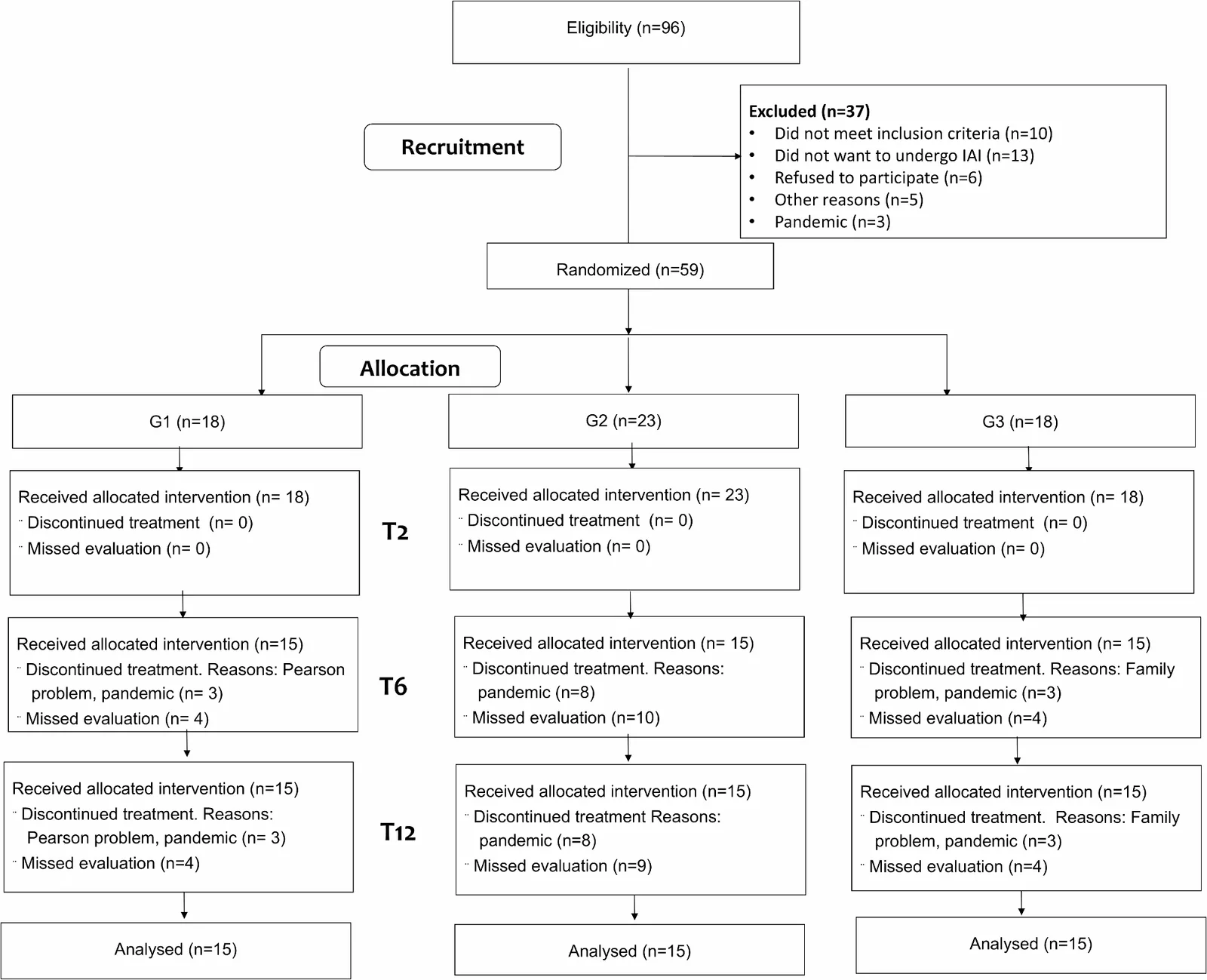
Flowchart of the study

A total of 96 elderly patients were deemed eligible; however, 37 were excluded for various reasons, as documented in the study flowchart following CONSORT guidelines (Fig. 2). The remaining 59 patients were randomized into three groups: 18 in the IIA/CE group (G1), 23 in the IIA/SS group (G2), and 18 in the IIA/placebo group (G3). Two patients withdrew from the study at T6—one in the IIA/placebo group due to family reasons and another in the IIA/CE group due to personal issues. Absences recorded until February 2020 were attributed to various reasons, including influenza, dental extractions, minor dermatological surgeries, falls, or personal matters. However, the onset of the COVID-19 pandemic in March 2020 significantly impacted patient recruitment and follow-up.

Initially, our study protocol included a 24-week follow-up assessment (T24). However, due to the substantial loss of patients caused by the pandemic, we opted to conduct an analysis of those who had completed the intervention up to T12 (completion of the PREP). Consequently, 15 patients were analyzed in each group. For those who missed assessments, their data were imputed using values from prior assessments.

### Adherence to PREP

The PREP intervention was designed as a 12-week program, with training sessions occurring twice per week, totaling 24 sessions. The mean attendance rate for the IAI/CE group was 87.75% (21.06 out of 24 sessions by T12), for the IAI/SS group it was 90.83% (21.8 out of 24 sessions by T12), and for the IAI/placebo group it was 83.33% (20 out of 24 sessions by T12). No adverse events related to the interventions were reported.

### Final sample characteristics

The final sample consisted of 45 elderly participants, 38 of whom were women (84.44%). A total of 34 participants (75.55%) self-identified as white. Radiographic classification using the Kellgren-Lawrence (KL) scale indicated that 22 participants (48.88%) had KL grade II, while 23 (51.11%) had KL grade III. Additionally, 13 participants (28.88%) were on continuous analgesic medication. Table 1 presents the clinical and demographic characteristics of the sample, demonstrating homogeneity across the groups in terms of demographic parameters, osteoarthritis-related characteristics, comorbidities, continuous medication use, and radiographic classification.

**Table 1:** Demographic and clinical characteristic at baseline

	IAI/CE + PREP	IAI/Saline + PREP	IAI/Placebo + PREP	
	(*n* = 15)	(*n* = 15)	(*n* = 15)	*p*
Gender (Women)	14 (93.3%)	13 (86.7%)	11 (73.3%)	0.463
Age (years)	70.5 ± 5.5	71.3 ± 5.6	71.9 ± 7.4	0.828
Education (years)	6.7 ± 3.4	6.7 ± 3.1	7.7 ± 3.2	0.627
Disease duration (years)	5.6 ± 2.4	6.1 ± 2.5	5.2 ± 1.8	0.467
BMI (kg/m^2^)	30.95 ± 5.42	29.31 ± 3.89	29.31 ± 4.61	0.548
Ethnicity (white)	9 (60.0%)	12 (80.0%)	13 (86.7%)	0.204
Marital status (maried)	9 (60.0%)	10 (66.7%)	9 (60.0%)	0.667
Caidor crônico	4 (26.7%)	2 (13.3%)	2 (13.3%)	0.697
Diseases				
Hypertension	10 (66.7%)	10 (66.7%)	12 (80.0%)	0.77
Diabetes Mellitus	8 (53.3%)	8 (53.3%)	4 (26.7%)	0.274
Dislipidemia	8 (53.3%)	10 (66.7%)	7 (46.7%)	0.651
Others	7 (17.5%)	8 (20.0%)	6 (15.0%)	0.841
Medications				
Hypertension	10 (66.7%)	10 (66.7%)	12 (80.0%)	0.77
Diabetes Mellitus	8 (53.3%)	9 (60.0%)	4 (26.7%)	0.176
Dislipidemia	8 (53.3%)	10 (66.7%)	7 (46.7%)	0.651
Oral analgesics	4 (26.7%)	5 (33.3%)	4 (26.7%)	1
NSAIDs	0 (0.0%)	0 (0.0%)	1 (6.7%)	1
Cartilage supplement	0 (0.0%)	0 (0.0%)	2 (13.3%)	0.318
Others	7 (46.7%)	7 (46.7%)	7 (46.7%)	0.318
Kellgren Lawrence grade				
II	8 (53.3%)	6 (40.0%)	8 (53.3%)	0.806
III	7 (46.7%)	9 (60.0%)	7 (46.7%)	0.806
Involvement				
Unilateral	5 (33.3%)	7 (46.7%)	3 (20.0%)	0.361
Bilateral	10 (66.7%)	8 (53.3%)	12 (80.0%)	0.361

### Primary outcome

Analysis of NPS scores (Table 2) revealed no statistically significant differences between the groups for either pain at rest (*p* = 0.800) or pain during movement (*p* = 0.142). However, a significant intragroup improvement over time was observed, as indicated by the 95% confidence interval (CI95%). All groups demonstrated reductions in pain from baseline (T0), both at rest (mean difference [CI95%]: -1.5 [-0.42 to 3.42] for IAI/CE; -3.7 [-5.73 to -1.66] for IAI/SS; -1.6 [-3.80 to 0.60] for IAI/placebo) and during movement (mean difference [CI95%]: -4.2 [2.49 to 5.90] for IAI/CE; -3.4 [-5.53 to -1.26] for IAI/SS; -3.0 [-4.99 to 1.00] for IAI/placebo).

**Table 2:**
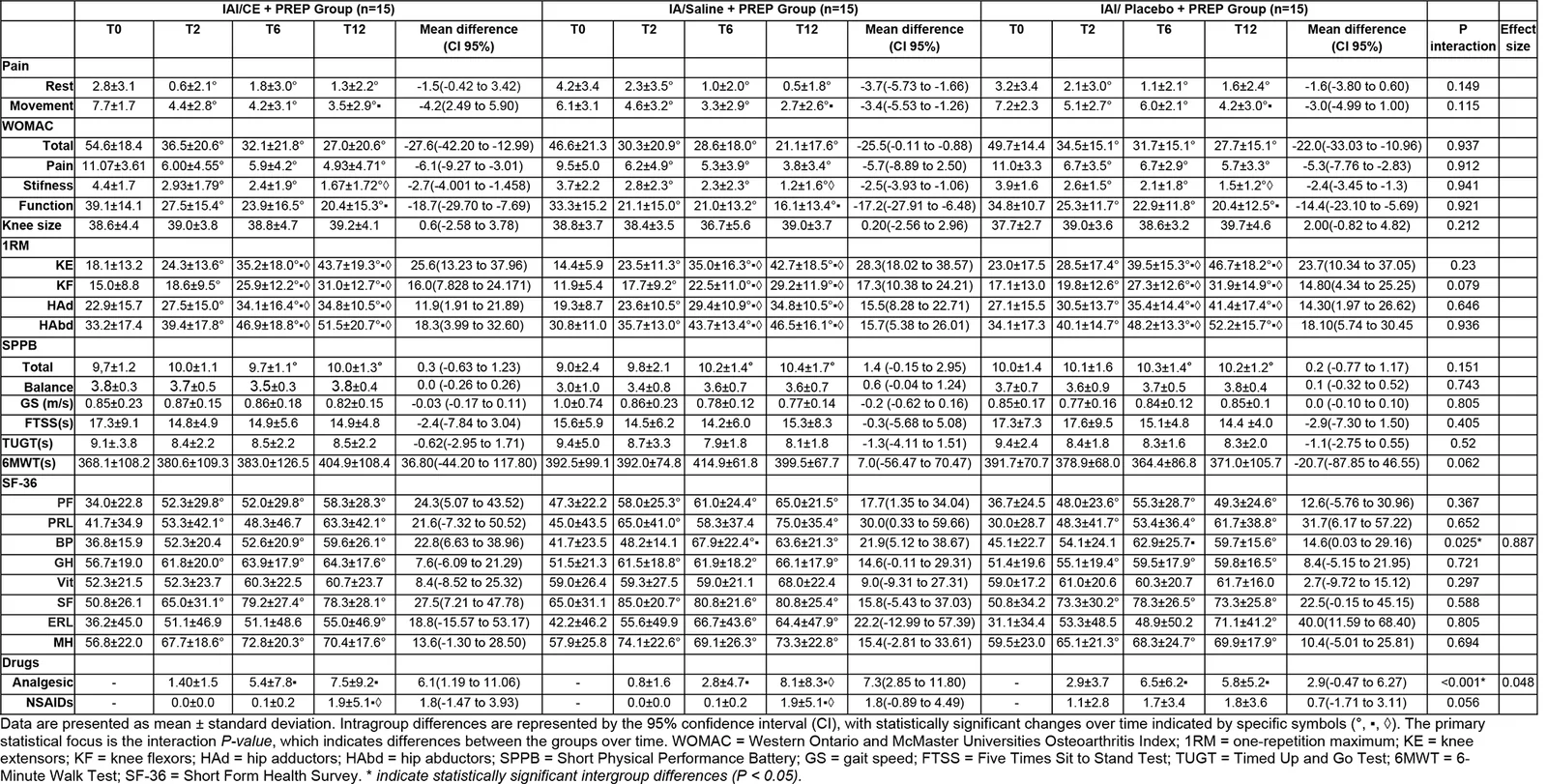
Changes over time and between-group comparisons for pain, physical function, and health outcomes across four evaluations

### Secondary outcomes

Between-group comparisons for secondary outcomes are presented in Table 2 for all evaluated parameters. No statistically significant differences were observed between the groups for the WOMAC domains of pain, stiffness, function, or total score (intergroup p-values: 0.436, 0.810, 0.540, and 0.588, respectively). However, all three groups exhibited statistically significant intragroup improvements over time (CI95%), indicating clinical benefits within each intervention group:

- WOMAC total score: mean difference [CI95%]: -27.6 [-42.20 to -12.99] for IAI/CE; -25.5 [-0.11 to -0.88] for IAI/SS; -22.0 [-33.03 to -10.96] for IAI/placebo.
- WOMAC pain domain: mean difference [CI95%]: -6.1 [-9.27 to -3.01] for IAI/CE; -5.7 [-8.89 to 2.50] for IAI/SS; -5.3 [-7.76 to -2.83] for IAI/placebo.
- WOMAC stiffness domain: mean difference [CI95%]: -2.7 [-4.001 to -1.458] for IAI/CE; -2.5 [-3.93 to -1.06] for IAI/SS; -2.4 [-3.45 to -1.3] for IAI/placebo.
- WOMAC function domain: mean difference [CI95%]: -18.7 [-29.70 to -7.69] for IAI/CE; -17.2 [-27.91 to -6.48] for IAI/SS; -14.4 [-23.10 to -5.69] for IAI/placebo.

Additionally, significant intragroup improvements were observed in muscle strength, assessed through the 1RM test, for knee extensors, knee flexors, hip adductors, and hip abductors (*p* < 0.001 for all groups), as demonstrated by the following mean differences and CI95%:

- Knee extensors: +25.6 [13.23 to 37.96] for IAI/CE; +28.3 [18.02 to 38.57] for IAI/SS; +23.7 [10.34 to 37.05] for IAI/placebo.
- Knee flexors: +16.0 [7.828 to 24.171] for IAI/CE; +17.3 [10.38 to 24.21] for IAI/SS; +14.8 [4.34 to 25.25] for IAI/placebo.
- Hip adductors: +11.9 [1.91 to 21.89] for IAI/CE; +15.5 [8.28 to 22.71] for IAI/SS; +14.3 [1.97 to 26.62] for IAI/placebo.
- Hip abductors: +18.3 [3.99 to 32.60] for IAI/CE; +15.7 [5.38 to 26.01] for IAI/SS; +18.1 [5.74 to 30.45] for IAI/placebo.

Improvements were also observed in the Short Physical Performance Battery (SPPB) (*p* = 0.007, valid for all three groups), with the following intragroup differences:

- SPPB total score: mean difference [CI95%]: +0.3 [-0.63 to 1.23] for IAI/CE; +1.4 [-0.15 to 2.95] for IAI/SS; +0.2 [-0.77 to 1.17] for IAI/placebo.

Regarding joint swelling (assessed via knee circumference in cm) and functional tests (TUGT and 6MWT), no statistically significant differences were found between the groups (*p* = 0.861, *p* = 0.992, and *p* = 0.616, respectively) or within groups over time (*p* = 0.148, *p* = 0.057, and *p* = 0.882, respectively).

The SF-36 questionnaire showed significant intragroup improvements across all domains except vitality (*p* < 0.001), with notable improvements in bodily pain (*p* = 0.025):

- Physical functioning: +24.3 [5.07 to 43.52] for IAI/CE; +17.7 [1.35 to 34.04] for IAI/SS; +12.6 [-5.76 to 30.96] for IAI/placebo.
- Bodily pain: +22.8 [6.63 to 38.96] for IAI/CE; +21.9 [5.12 to 38.67] for IAI/SS; +14.6 [0.03 to 29.16] for IAI/placebo.

Analgesic consumption increased over time (*p* < 0.001), potentially influencing observed improvements. The IAI/CE group exhibited a significant increase in analgesic use between T2 and T12 (*p* = 0.013), though no significant differences were found between the groups at any time point (*p* = 0.065 at T2, *p* = 0.110 at T6, *p* = 0.684 at T12).

Finally, based on Table 3, which assesses patient satisfaction with the treatment, we observed variations in participants’ perceptions over the 12-week period. In the IAI/CE + PREP group, most participants reported improvement over time, with 66.7% indicating they felt “much better” at T2 and T6, although this proportion decreased to 46.7% at T12. In the IAI/Saline + PREP group, responses were more varied, with a progression in the feeling of “much worse” and “a little worse” over time, suggesting a less favorable perception of the treatment. The IAI/Placebo + PREP group showed a more balanced distribution of responses, with 60% of participants reporting worsening well-being perception at T12. Statistical analysis did not show significant differences between groups (*p* = 0.335), indicating that patient satisfaction with the treatments followed similar patterns regardless of the intervention applied. These findings reinforce the importance of non-pharmacological approaches in patients’ perceived improvement in knee osteoarthritis.

**Table 3:** Assessment patient satisfaction with the treatment

Likert scale		IAI/CE + PREP group (*n* = 15)			IAI/Saline + PREP group (*n* = 15)			IAI/Placebo + PREP group (*n* = 15)			P*
		T2	T6	T12	T2	T6	T12	T2	T6	T12	
1		0 (0.0)	0 (0.0)	0 (0.0)	1 (6.7)	0 (0.0)	0 (0.0)	0 (0.0)	0 (0.0)	0 (0.0)	0.335
2		1 (6.7)	1 (6.7)	2 (13.3)	0 (0.0)	0 (0.0)	0 (0.0)	0 (0.0)	0 (0.0)	1 (6.7)	
3		1 (6.7)	1 (6.7)	0 (0.0)	3 (20.0)	2 (13.3)	1 (6.7)	7 (46.7)	4 (26.7)	1 (6.7)	
4		3 (20.0)	3 (20.0)	6 (40.0)	7 (46.7)	5 (33.3)	5 (33.3)	3 (20.0)	5 (33.3)	9 (60.0)	
5		10 (66.7)	10 (66.7)	7 (46.7)	4 (26.7)	8 (53.3)	9 (60.0)	5 (33.3)	6 (40.0)	4 (26.7)	

*P = ANOVA (analysis of variance for repeated measures) over time; T0 = baseline; T2 = evaluation after 2 weeks; T6 = evaluation after 6 weeks; T12 = evaluation after 12 weeks; 1: I feel much better; 2: I feel a little better; 3: I feel like before; 4: I feel a little worse or 5: I feel much worse

## Discussion

This study aimed to evaluate the effectiveness of prior intra-articular corticosteroid injection (IAI/CE) compared to saline (IAI/SS) and placebo in elderly patients with knee osteoarthritis (OA) undergoing a progressive resistance exercise program (PREP). After 12 weeks, no statistically significant differences were observed between groups regarding pain, functional performance, or quality of life. However, all groups exhibited significant intragroup improvements over time.

The lack of superiority of intra-articular corticosteroid injection over saline or placebo may be explained by several physiological mechanisms. One possibility is that the transient anti-inflammatory and analgesic effects of corticosteroids were insufficient to provide sustained benefits when combined with a structured exercise program. Additionally, the phenomenon of ‘regression to the mean’ in pain scores, commonly observed in osteoarthritis studies, may have contributed to similar improvements across groups.

Another explanation is that the exercise intervention itself, by promoting neuromuscular adaptations and reducing joint load, might have exerted a predominant effect on pain and function, thereby masking potential differences between treatments. Future studies incorporating biomarkers of inflammation and imaging assessments of synovial changes could provide further insights into the interactions between intra-articular treatments and exercise therapy.

With the aging global population, symptomatic knee OA is estimated to affect at least 4% of individuals. However, its actual prevalence may be underestimated, as diagnosis typically occurs after symptom onset. This condition is particularly prevalent among those over 50 years old, reaching 50% in individuals above 80 [33]. Knee OA has a substantial societal impact, accounting for over 90% of total knee arthroplasty indications [34].

The improvements observed across all groups reinforce the role of PREP in knee OA management. This finding aligns with previous research highlighting exercise as a key therapeutic strategy, regardless of adjunct intra-articular treatments [6, 7, 23]. Similarly, Henriksen et al. [14] found that administering IAI/CE before an exercise program did not enhance outcomes compared to saline injection. Moreover, a study by Guvendi et al. [15] reported that IAI/CE provided inferior pain relief compared to platelet-rich plasma (PRP), suggesting that corticosteroid injections may have limited benefits when combined with structured resistance training.

Aging-related biological changes contribute to a chronic low-grade pro-inflammatory state, playing a key role in OA pathogenesis. This phenomenon, known as inflamm-aging, results from cumulative antigenic exposure and various stressors that modulate immune responses, leading to immunosenescence [35]. Based on this context, we hypothesized that reducing knee inflammation through a corticosteroid injection before exercise could enhance its benefits. However, our results suggest that structured resistance training alone was sufficient to promote improvements in pain and function, regardless of prior intra-articular treatment.

Functional performance was assessed using validated tests commonly employed in elderly populations and individuals with knee OA. These included the Timed Up and Go Test (TUGT), the Short Physical Performance Battery (SPPB), and the Six-Minute Walk Test (6MWT). Baseline TUGT and SPPB scores indicated good physical performance, with no increased risk of falls or probable sarcopenia, as evidenced by TUGT times below 10 s and SPPB scores exceeding 8 points [36]. Although all groups showed improvements in SPPB scores over time, these changes were not clinically relevant, as participants maintained moderate to good physical performance throughout the study.

Quality of life is often impaired in knee OA patients due to pain and functional limitations. In our study, this was assessed using the SF-36 questionnaire. The findings demonstrated improvements across all domains except vitality, corroborating previous studies [37–40]. However, prior research has predominantly focused on physical function and mental health [38–40], with limited investigations incorporating IAI/CE. These results further reinforce the role of structured exercise programs in improving overall well-being, regardless of intra-articular treatment.

Despite the expected short-term analgesic effects of IAI/CE, no significant between-group differences in pain reduction were observed. This suggests that factors beyond the injected substance contributed to clinical improvements. One possible explanation is the documented therapeutic effect of saline injections, which have been shown to provide symptomatic relief [16, 17]. Moreover, the placebo group exhibited similar improvements, highlighting the role of patient expectations and the structured nature of the intervention. These findings align with previous studies indicating that intra-articular injections, regardless of the substance used, may elicit an analgesic response due to the procedural effect [17].

Knee swelling was evaluated to assess short-term IAI effects. However, no significant reduction in knee circumference was observed in the IAI/CE group. Previous studies conducted by our research group have used this method [41, 42], but it may lack the sensitivity to detect subtle intra-articular changes. Future research incorporating ultrasound-based assessments could provide a more precise evaluation of joint effusion and inflammation in response to different treatments.

Muscle strength, assessed through the one-repetition maximum (1RM) test, improved in all groups, reinforcing the benefits of progressive resistance training. The absence of adverse effects allowed for proper training progression, while individualized exercise programs likely enhanced adherence and motivation. These findings align with the European League Against Rheumatism (EULAR) recommendations, which advocate for personalized assessments and tailored interventions in OA management [6].

Comparative studies on intra-articular treatments suggest that the type of injectable agent may influence functional outcomes. While IAI/CE did not outperform saline or placebo in our study, alternative agents such as PRP [43], botulinum toxin [44], and hyaluronic acid [45] have demonstrated potential benefits when combined with exercise-based rehabilitation. Future research should explore whether different injectables yield superior outcomes when integrated into structured rehabilitation programs.

Lastly, increased analgesic use at T2 may have influenced pain scores. However, since analgesic consumption did not differ significantly between groups, it is unlikely that medication use was the primary driver of pain relief. Future studies should implement stricter analgesic control to better isolate the effects of intra-articular treatments. Nonetheless, it is important to acknowledge that the overall increase in analgesic consumption over time may have contributed to the observed pain reduction in all groups. Although no significant differences between groups were detected, the progressive increase in medication use could have acted as a confounding factor, potentially masking subtle interventional effects. Future research should consider stricter analgesic control and alternative statistical approaches to better account for the impact of rescue medication on clinical outcomes.

### Study limitations

This study has several limitations. The small sample size, constrained by recruitment challenges during the COVID-19 pandemic, may have reduced the statistical power to detect between-group differences. Although our study was adequately powered for intragroup analyses, the reduced sample size likely impacted the ability to detect subtle differences between groups, particularly for secondary outcomes. The limited statistical power increases the risk of type II error, meaning that true differences between interventions may not have been identified. Future studies with larger cohorts and improved recruitment strategies are needed to provide more robust estimates of treatment effects.

The follow-up period was limited to 12 weeks, preventing the assessment of long-term outcomes and potential symptom recurrence after corticosteroid injection. Since intra-articular corticosteroids typically provide only transient benefits, extended follow-up studies are necessary to evaluate their long-term effects. Additionally, this study did not incorporate imaging techniques, such as ultrasound, to quantify joint inflammation or synovitis. Previous research suggests that corticosteroid injections may be more effective in patients with active inflammation. Therefore, the lack of imaging assessments may have limited our ability to distinguish responders from non-responders. Future studies should consider integrating objective biomarkers to improve patient stratification.

Although medication use was monitored, individual variations in pain perception and adherence to the exercise protocol could have influenced the outcomes. While attendance rates were high across all groups, more precise tracking methods—such as wearable activity monitors—could provide more detailed compliance data and insights into physical activity levels outside structured interventions.

Finally, administering a single corticosteroid injection one week before exercise training may not fully reflect clinical practice, where repeated injections or different timing strategies are often employed. Future research should explore the potential benefits of multiple injections or alternative treatment sequences in combination with exercise therapy to optimize outcomes in elderly patients with knee OA.

## Conclusion

The combination of IAI/CE and a 12-week progressive resistance training program did not demonstrate superiority over IAI/saline or placebo when associated with the same exercise regimen in improving pain, function, and quality of life in elderly patients with knee OA.

## Data Availability

No datasets were generated or analysed during the current study.
